# Temperament Types at Age 3 and Smartphone Overdependence at Age 10

**DOI:** 10.3389/fpsyg.2022.833948

**Published:** 2022-03-10

**Authors:** Yeon Ha Kim

**Affiliations:** Department of Child and Family Studies, Kyung Hee University, Seoul, South Korea

**Keywords:** temperament type, cluster analysis, longitudinal, overdependence, smartphone

## Abstract

Few studies have addressed the longitudinal links between early temperament types and later problematic smartphone use. This study aims to identify children’s early temperament types at age 3 and to examine the link between the temperament types and smartphone overdependence at age 10. This study utilized a population-based data set presented by the Panel Study on Korean Children. Based on emotionality, activity, and sociability levels at age 3, children were clustered into similar temperament types. Links between the early temperament types and the risks of smartphone overdependence at age 10 were identified through analyses of covariances and binary logistic regressions. Three early temperament types were identified among Korean children: reactive (28.1%), sociable (37.2%), and cautious (34.8%). Children’s smartphone dependence at age 10 differed according to the temperament types identified at age 3. Compared to children with the sociable temperament type, children with the reactive type or the cautious type had an increased risk of smartphone overdependence. The link between temperament types at age 3 and smartphone overdependence at age 10 was meaningful. The cautious children were the most vulnerable group to the risk of smartphone overdependence. Temperament type identification in early years may be a useful measure for screening groups of children who are at risk for problematic smartphone use and need proactive interventions.

## Introduction

Smartphones play an important role in our daily lives. Along with the rapid development of mobile technology, we ubiquitously access the Internet using portable smart devices and experience various media services via online platforms. Along with the prevalence of smartphones, the concern for problematic smartphone use has risen not only for adolescents and adults but also for young children. A recent meta-analytic review based on data of 24 countries reveals that problematic smartphone use is increasing across the world (Olson et al., 2022). According to an investigation in Korea (Ministry of Science and ICT & National Information Society Agency, 2020), over 23% of smartphone users are at risk of smartphone overdependence, and the overdependence rate of the younger generation is higher than that of the total population: 27.3% of children ages 3 to 9 and 35.8% of those ages 10 to 19 are overdependent on smartphones (Ministry of Science and ICT & National Information Society Agency, 2020).

Sin et al. (2011) explain smartphone overdependence by three states: increased salience, self-control failure, and experiencing serious consequences). Increased salience indicates that a smartphone is prominent in an individual’s daily activities, and the individual considers using a smartphone as the most important activity in which they engage. Self-control failure means that the individual fails to regulate smartphone usage as measured against their personal goals. Experiencing serious consequences indicates that the individual keeps using a smartphone despite experiencing negative physical, psychological, and social consequences caused by excessive smartphone use. For young children at risk of smartphone overdependence, increased salience is more distinct than in other age groups, which indicates that they spend most of their time using smartphones and prioritize finding opportunities for smartphone use (Ministry of Science and ICT & National Information Society Agency, 2020). Considering that young children need various cognitive, social, and physical activities for healthy development, problematic smartphone use may hinder their developmental potential.

Many studies have tried to investigate the predictors of problematic smartphone use. So far, demographic factors (i.e., gender and age), social family factors (i.e., peer relationships, family functions, and parenting), personality factors (i.e., self-esteem and personality traits), and smartphone usage patterns (i.e., duration of use and preferred activities) have been explored (Lee et al., 2016; Fischer-Grote et al., 2019; Ministry of Science and ICT & National Information Society Agency, 2020). However, most of these studies are cross-sectional, so that cause-and-effect relationships between variables may be obscured. Also, studies focusing on young children’s problematic smartphone use from a longitudinal perspective are extremely rare.

Meanwhile, temperament traits in the early years have gained special attention because of the strong relationship between these traits and later mental health and psychopathology (Kostyrka-Allchorne et al., 2020). Temperament indicates individual differences in reactions to external stimuli and self-regulation. Temperament traits are fairly stable, and individual differences can be observed at a very early age (Planalp and Goldsmith, 2020; Takács et al., 2020). So far, many studies have reported meaningful links between early temperament and the later emotional and behavioral functions of children (Gartstein et al., 2012; Kostyrka-Allchorne et al., 2020; Kozlova et al., 2020; Tang et al., 2020). However, few studies have explored the longitudinal link between early temperament and later problematic smartphone use. This is due mainly to smartphone overdependence, a newly emergent mental health issue, which has seldom been included as a variable in longitudinal data collection.

In Korea, which has the top smartphone ownership rate in the world, the younger generation’s problematic smartphone use has become a major social concern. The Korean government conducts an annual national survey on smartphone overdependence (Ministry of Science and ICT & National Information Society Agency, 2020), and several longitudinal data collection projects supported by the Korean government have investigated children’s smartphone usage. Thus, accumulated longitudinal data exploring the links between early temperament and later smartphone overdependence among Korean children is now available.

When examining the relationship between temperament and mental health issues, there are two possible analytic approaches. The first approach is the variable-centered approach, which means that the sub-dimensions of temperament are simultaneously entered into the analytical plan as independent variables which verify the significance of predicting power (Gartstein et al., 2012; Kostyrka-Allchorne et al., 2020). The second approach is the person-centered approach (Sanson et al., 2009; Prokasky et al., 2017). Since an individual’s temperament is a unique combination of sub-dimensions, people are classified into temperament types that share similar intrapersonal differences with the sub-dimensions. Then the differences in mental health risks based on temperament type are explored (Sanson et al., 2009; Dalimonte-Merckling and Brophy-Herb, 2019).

The present study will take the person-centered approach for two reasons. First, the person-centered approach enables a comprehensive understanding of how the combinations of sub-dimensions of temperament jointly work when they are linked to a specific outcome variable. For example, Buss and Plomin (1984) proposed early temperament traits such as emotionality, activity, and sociability. Emotionality indicates children’s reactions to stimuli with emotional distress such as fear, irritation, frustration, and sadness. Activity is the level of energy children show in physical movement both in terms of rate and extent. Sociability indicates children’s preferences to be in the company of others as compared to being alone. In temperament research, high emotionality has mostly been a risk factor for both external and internal problems (Kostyrka-Allchorne et al., 2020; Kozlova et al., 2020). High activity has been a risk factor for externalizing problems (Kostyrka-Allchorne et al., 2020) but also a protective factor against internalizing problems (Gartstein et al., 2012; McDowell et al., 2017). Low sociability has often been associated with internal problems such as anxiety or depression (Tang et al., 2020). However, with this variable-centered information, it is hard to predict whether or not children with a high emotionality and high activity combination might differ from children with a high emotionality and low activity combination in their risk for developing a specific mental health issue. An individual’s function is the result of dynamic interactions between multiple intrapersonal traits and abilities. Clustering children sharing similar temperamental profiles and examining the differences between the clusters can more precisely address the link with smartphone overdependence.

Second, the person-centered approach gives us typological information as to which specific type might have greater potential risk for a particular mental issue. If we are able to explain the risk of smartphone overdependence to parents and practitioners using typological examples, they will be better able to understand and prepare to help children. Identifying temperament or personality types that are vulnerable to specific health issues is not an entirely new approach. For example, Type D personality is characterized by social inhibition accompanied by negative affectivity (Denollet, 2000, 2005). People with Type D personality are known to be vulnerable to many mental health issues (Denollet, 2000). A recent study reported that Chinese adolescents’ Type D personalities were positively linked with addictions to online social networking sites and the restorative outcomes from social networking sites enhanced the links (Nie et al., 2019). Kim (2021) investigated whether Korean children’s media use at age 9 differed by the combinations of emotionality and activity observed in early years. Children with a high negativity and low activity combination were the most vulnerable to problematic media use. Well-defined typological information on children’s temperament types and the potential links with specific mental health issues may be an easy and effective way to close the gap between research and practice.

Children’s temperament is culturally bound, and the socio-emotional functions of each temperament type should be understood in proper cultural contexts (Chen, 2018). In the New York Longitudinal Study, Thomas and Chess classified children’s temperaments into the difficult, easy, and slow to warm up types (Thomas and Chess, 1977, 1986). Prokasky and colleagues identified six temperament types among children in the United States: unregulated, regulated, high reactive, bold, average, and well-adjusted (Prokasky et al., 2017). Sanson and colleagues reported four clusters of early temperament types in Australian children: reactive/inhibited, poor attention regulation, non-reactive/outgoing, and high attention regulation (Sanson et al., 2009). Planalp and Goldsmith (2020) identified four temperament types among infants in the United States: typical, low negative, withdrawn/inhibited, and positive/active or low reactive. Lin et al. (2021) profiled three temperament profiles of infants from Mexican American families; the high positive affect, well-regulated profile, the negative reactive, low regulated profile, and the low positive affect, low regulated profile. Differences in the temperament types that appeared in the previous studies are expected because their temperament measurements, populations studied, and analytic approaches varied. Identifying culturally representative temperament types and understanding the uniqueness of each type is critical. With advanced analytical methods and nationally representative data, temperament clusters representing the Korean context need to be identified and used to anticipate the future adjustment needs of children.

In summary, the present study intends to investigate the longitudinal link between early temperament types and later problematic smartphone use. Using data from the Panel Study on Korean Children, a nationwide population sample, early temperament types of Korean children (at age 3) will be identified. Also, associations between early temperament type and smartphone overdependence at age 10 will be investigated. It is expected that children will be clustered into several groups sharing similar profiles of emotionality, activity, and sociability traits. Also, it is expected that children with a specific type of early temperament may be more vulnerable to problematic smartphone use as compared to their peers.

## Materials and Methods

### Participants

The Panel Study on Korean Children (PSKC) is a nationwide data collection project conducted by the Korea Institute of Child Care and Education. Since 2008, the PSKC has annually released comprehensive information on child development, parenting, and family functioning in Korean households. A total of 2,150 homes were sampled using a stratified multi-stage sampling method. The sample retention rate when children reached age 3 was 81.6%, and age 10 was 66.7%. The characteristics of participants are presented in Table 1.

**TABLE 1:** Characteristics of participants at age 10 (*n* = 1296).

‘	Temperament types at age 3						Total		Statistic Indices
	Reactive 29.2%		Sociable 34.0%		Cautious 36.7%			
	M (SD)	%	M (SD)	%	M (SD)	%	M (SD)	%	
Child sex (male)		53.8		51.4		53.4		50.5	χ*^2^* = 4.059 *df* = 2 *N* = 1294
Maternal education (2-year college or above)		73.9		75.7		71.9		73.9	χ*^2^* = 1.729 *df* = 2 *N* = 1282
Paternal education (2-year college or above)		71.4		72.5		74.4		72.9	χ*^2^* = 0.971 *df* = 2 *N* = 1286
Employed mothers		55.2		61.6		56.9		58.1	χ*^2^* = 3.868 *df* = 2 *N* = 1261
Maternal daily stress	3.02(0.648)		2.84(0.632)		2.96(0.656)		2.94(0.649)		*F*_(2_,_1293)_ = 9.147 *P* = 0.000
Maternal life satisfaction	3.41(0.816)		3.60(0.693)		3.39(0.764)		3.47(0.760)		*F*_(2_,_1293)_ = 10.892 *P* = 0.000
Maternal subjective health	3.32(0.726)		3.47(0.741)		3.30(0.717)		3.37(0.732)		*F*_(2_,_1293)_ = 7.490 *P* = 0.001
Family monthly income (Korean won)	535.39 (468.437)		582.06 (570.093)		548.47 (413.298)		556.89 (491.085)		*F*_(2_,_1169)_ = 0.948
Smartphone use at age 10	25.029 (6.586)		23.769 (6.0651)		25.104 (6.176)		24.592 (6.285)		*F*_(2, 1293)_ = 6.517 *p* = 0.000
At risk of smartphone overdependence at age 10		34.0		25.4		36.7		31.8	χ*^2^* = 14.762 *df* = 2 *N* = 1296 *p* = 0.001
At high risk of smartphone overdependence at age 10		26.4		20.8		28.1		24.9	χ*^2^* = 7.167 *df* = 2 *N* = 1296 *P* = 0.028

### Measures

#### Temperament at Age 3

The Korean version of the Emotionality, Activity, and Sociability Temperament Questionnaire (EAS) (Buss and Plomin, 1984; Mathiesen and Tambs, 1999; Korea Institute of Childcare and Education, 2021) was employed to measure children’s emotionality, activity, and sociability levels at age 3. Mothers rated their child’s traits from 1 (not characteristic or typical of your child) to 5 (very characteristic or typical of your child). The EAS developed by Buss and Plomin (1984) measures temperament of children aged 1 to 9 with four sub-factors (emotionality, activity and shyness and sociability). The PSKC research team revised the EAS with three sub-factors (emotionality, activity, and sociability) by combining sociability and shyness items. The internal consistencies (Cronbach’s alpha) of the emotionality (5 items; e.g., “React intensely when upset”), activity (5 items; “Is very energetic”), and sociability (10 items; “Likes to be with people”) scales were.725,0.781, and.831, respectively.

#### Smartphone Overdependence in Children

Children’s smartphone overdependence was measured with the Internet Addiction Proneness Scale for Youth: Observer-report (Sin et al., 2011). This instrument consists of 15 items (e.g., “He or she gets in trouble with his/her family because of his/her Internet usage”; “Unlike his/her usual self, he/she appears confident and outspoken when he/she is on the Internet.”). Observers can rate children’s smartphone behaviors from 1 (strongly disagree) to 4 (strongly agree). The PSKC research team revised the items by changing the term ‘Internet’ to ‘PC, smartphone’ to include comprehensive behaviors across various Internet devices. Mothers were asked to rate their children’s smartphone use at age 10. The cutoff score for elementary students who are at risk of smartphone overdependence is 28, and 30 is the cutoff score for elementary students who are at high risk of smartphone overdependence. In this study, the at-risk group includes children with a score of 28 or more; the high-risk group includes children with a score of 30 or more. The internal consistency (Cronbach’s alpha) of the 15 items in this study was.855.

### Analyses

Children’s temperament types at age 3 were identified using a two-step cluster analysis: hierarchical cluster analysis and K-means cluster analysis. To examine the differences in children’s smartphone use at age 10 by their temperament type at age 3, analyses of covariance and Bonferroni multiple comparisons were used. Two-step binary logistic regressions were conducted to identify the odds of being at risk or at high risk of smartphone overdependence according to temperament clusters.

Participants’ demographic characteristics such as child sex, parents’ education, maternal employment status, and family monthly income were controlled when analyzing the differences in children’s smartphone use by their temperament types. Mothers’ psychological conditions (maternal daily stress, life satisfaction, and subjective health condition) at child age 10, which possibly affected ratings on their children’s smartphone use, were also controlled. A single question was employed for each condition (e.g., how much are you stressed on a daily basis?) and mothers rated their own conditions with a five-point Likert scale (e.g., not at all = 1, somewhat = 3, extremely = 5).

## Results

### Temperament Type at Age 3

**FIGURE 1:**
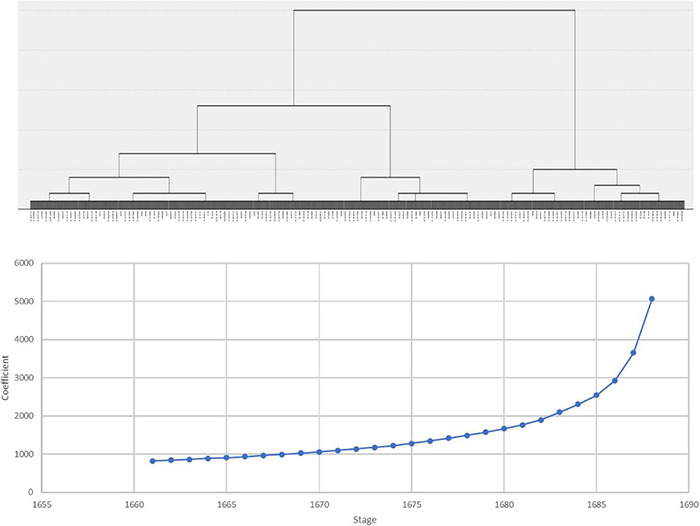
Dendrogram and agglomeration schedule of hierarchical clustering analysis.

A total of 1,689 children’s emotionality, activity and sociability scores at age 3 were standardized into Z-scores. Using the Z scores as clustering variables, a hierarchical clustering analysis using the Ward linkage method and squared Euclidean distance was conducted. Figure 1 presents the dendrogram and the agglomeration schedule of hierarchical clustering analysis. The dendrogram and agglomeration schedule coefficients indicate that the three-cluster model might best describe Korean children’s temperament types at age 3. A K-means cluster analysis with three clusters followed. As presented in Table 2, the three clusters are properly differentiated from each other.

**TABLE 2:** Three clusters of temperament at age 3 (*n* = 1689).

Center of cluster	Reactive (*N* = 474, 28.1%)	Sociable (*N* = 628, 37.2%)	Cautious (*N* = 587, 34.8%)	*F*_(2, 1686)_
Emotionality	0.79031	–0.869	0.269	775.637 *p* = 0.000
Activity	0.71182	0.325	–0.918	775.849 *p* = 0.000
Sociality	0.3306	0.603	–0.902	682.400 *p* = 0.000

**FIGURE 2:**
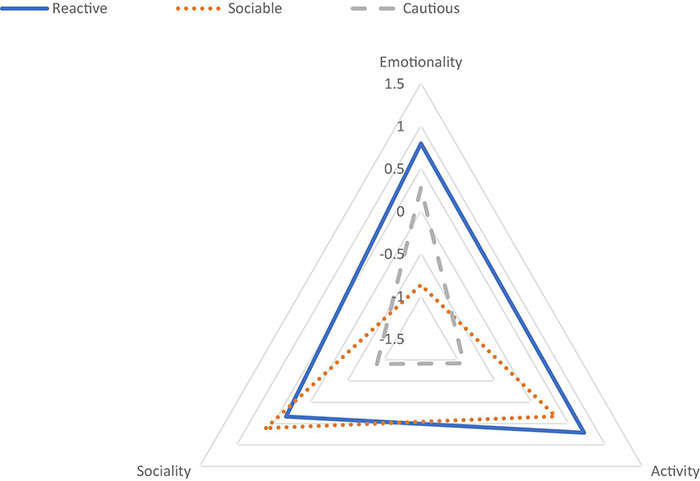
Center of clusters of three types of temperament at age 3.

Figure 2 graphically depicts how the three types of temperament are distinct from each other in terms of the center of the clusters. The first type of temperament (28.1%) has high emotionality and activity and average sociability. These children can be described as *reactive* because they intensely respond to external stimuli as well as being psychically active in both rate and intensity. The second cluster (37.2%) has the lowest level of emotionality and the highest level of sociability among the three clusters. The activity level was average. The children can be described as *sociable* because they like being with others and approach new people or situations without frustration. The last cluster (34.8%) is characterized by low activity and sociability levels, which were close to -1 SD, with average emotionality. These children can be described as *cautious* because they are careful when encountering new social situations and reluctant to physically respond to stimuli.

### Smartphone Use of Children at Age 10 by Temperament Type at Age 3

Among the 1,689 children whose temperament types at age 3 were identified, 1,296 data on children’s smartphone use at age 10 were available. As Table 3 presents, the main effects of temperament types at age 3 were statistically valid in predicting smartphone use at age 10. The *post hoc* comparison results revealed that (Table 4) children in the sociable cluster were less likely to use smartphone than children in the reactive cluster and in the cautious cluster. There was no statistically meaningful difference in smartphone use between the reactive cluster and the cautious cluster.

**TABLE 3:** Main effects of temperament types at age 3 on smartphone use at age 10.

Variables	*F*	Partial η^2^	Probability
Child sex (male)	44.698	0.038	0.000
Maternal education (2-year college or above)	14.412	0.013	0.000
Paternal education (2-year college or above)	2.252	0.002	0.134
Employed mothers	6.549	0.006	0.011
Maternal daily stress	1.824	0.002	0.177
Maternal life satisfaction	17.316	0.015	0.000
Maternal subjective health	0.002	0.000	0.963
Family monthly income (Korean won)	5.512	0.005	0.019
Temperament type at age 3	7.217	0.013	0.001
		*R*^2=^ 0.114	

**TABLE 4:** Smartphone use at age 10 by temperament types at age 3.

		M	SE	Group Comparisons
	Reactive	24.811	0.328	Sociable < Reactive*, Cautious**
	Sociable	23.624^a^	0.296	
	Cautious	25.174^a^	0.301	

Adjusted variables are child sex, maternal education, paternal education, employed mothers, maternal daily stress, maternal life satisfaction, maternal subjective health and family monthly income. *p < 0.05.

**p < 0.01.

### Risk of Smartphone Overdependence at Age 10 by Temperament Type at Age 3

As Table 5 presents, meaningful differences were observed in the risk of smartphone overdependence at age 10 according to temperament types at age 3. Compared to children in the sociable cluster, children in the reactive cluster and children in the cautious cluster had increased odds of being at risk for smartphone overdependence. Compared to children in the sociable cluster, children in the cautious cluster had increased odds of being at high risk for smartphone overdependence.

**TABLE 5:** Smartphone overdependence at age 10 by temperament clusters at age 3.

Variables	At risk			At high risk		
	Walds	Probability	Exp(B)	Walds	Probability	Exp(B)
Child sex (boys as reference)	29.713	0.000	0.472	20.382	0.000	0.516
Maternal education (2-year college or above as reference)	13.881	0.000	1.919	6.091	0.014	1.582
Paternal education (2-year college or above as reference)	0.414	0.520	1.119	0.346	0.557	1.115
Employed mother	5.036	0.025	1.373	5.501	0.019	1.426
Maternal daily stress	0.955	0.329	1.126	1.069	0.301	1.144
Maternal life satisfaction	11.098	0.001	0.703	14.654	0.000	0.652
Maternal subjective health	1.346	0.246	0.891	0.015	0.903	1.013
Family monthly income (Korean won)	3.414	0.108	1.000	5.790	0.016	1.000
Temperament type (Sociable type as reference)						
Reactive	7.191	0.007	1.592	3.293	0.070	1.400
Cautious	15.639	0.000	1.921	8.208	0.004	1.654

## Discussion

This study was conducted with two specific goals. The first goal was to identify early temperament types among Korean children. For this, a two-step clustering analysis was conducted with emotionality, activity, and sociability levels of children at age 3 as clustering variables. The second goal was to examine if the temperament types were linked to later smartphone overdependence. For this, children’s smartphone usage at age 10 was compared by early temperament types using analyses of covariance and binary logistic regression analyses. Here the researcher presents those findings and implications.

First, Korean children at age 3 were grouped into three distinct temperament groups: reactive (28.1%), sociable (37.2%), and cautious (34.8%). Among the three early temperament types in this study, the reactive type was distinct from the other groups with high emotionality and activity. The reactive type is comparable to the unregulated type from Prokasky and colleagues for high activity and emotionality (Prokasky et al., 2017). Children of this type often show intense reactions to external stimuli and are characterized as difficult children (Thomas and Chess, 1977, 1986). They have been described as the most challenging type for parents and teachers to deal with (Thomas and Chess, 1986; Bao et al., 2016; Prokasky et al., 2017; Dalimonte-Merckling and Brophy-Herb, 2019).

The sociable type was characterized by high sociability and low emotionality which is comparable to the non-reactive/outgoing type from Sanson and colleagues (Sanson et al., 2009). The sociable children are in a positive mood most of the time and prefer interacting with others to being alone, which are often characterized as easy children (Thomas and Chess, 1977, 1986). The sociable children are expected to have good relationships with others and to be well adjusted at school. In Sanson and colleagues’ study, both parents and teachers rated the non-reactive/outgoing type of children as having better social and academic skills as compared to children with other temperament types (Sanson et al., 2009).

The cautious type displayed the lowest activity and sociability levels among the three types. Though the activity and sociability levels of the cautious type were close to -1 SD; the emotionality level was above the mean (0.269 SD). The cautious children are similar to the regulated children from Prokasky and colleagues with low activity and sociability (Prokasky et al., 2017), and the withdrawn/inhibited children from the profiles of Planalp and Goldsmith (2020) in terms of low activity, positive affection, and interest. They are also comparable to the reactive/inhibited children described by Sanson and colleagues in demonstrating large intrapersonal differences between emotionality and sociability (Sanson et al., 2009). The cautious children may look well behaved because of their restrained social and physical behaviors, but their emotional distress may be substantial. In Sanson and colleagues’ study, the reactive/inhibited children displayed more parent-reported anxiety and attention problems and poor social skills as compared to their peers (Sanson et al., 2009). Socio-behavioral inhibition observed in the early years is a specific risk factor for internalizing problems in later years (Katz et al., 2011; Tang et al., 2020). Tang and colleagues reported that infants with higher levels of cautious and fearful behaviors became more reserved and introverted adults with lower social functioning as compared to their peers (Tang et al., 2020).

Second, there was a significant main effect of temperament types at age 3 on smartphone use at age 10. The main effect of temperament types was significant when participants’ demographic characteristics (child sex, parents’ education, maternal employment status, family monthly income) and the mothers’ psychological conditions (maternal daily stress, life satisfaction, and subjective health) were controlled. The smartphone use of the sociable children was significantly lower than that of the other two types of children. The well-known typological information that children with sociable and easy temperamental characteristics are less likely to experience mental health issues (Sanson et al., 2009; Bao et al., 2016; Dalimonte-Merckling and Brophy-Herb, 2019) is also applicable to problematic smartphone use.

Additional analyses were conducted to examine which type of temperament had a greater risk of smartphone overdependence. Regarding the risk of smartphone overdependence (a total score of 28 or more), the reactive type had over one and a half times increased chance of being at risk for smartphone overdependence as compared to the sociable type. The cautious type was almost two times more likely to be at risk of smartphone overdependence than the sociable type. Regarding the high risk of smartphone overdependence (a total score of 30 or more), the cautious type was over one and a half times more likely to be at high risk of smartphone overdependence than the sociable type.

Certainly, the current findings indicate that the cautious type is the most vulnerable to the risk of smartphone overdependence among the three types of temperament. Unexpectedly, the reactive type, displaying the highest emotionality and activity among the three types, was not the most vulnerable group to smartphone overdependence. In previous research, these children have been considered the most difficult type because of the under-controlled external behaviors (Bao et al., 2016). Why does the cautious type have a greater risk of problematic smartphone use than the other types? It should be noted that the cautious children display significant intrapersonal differences between emotionality and the other two temperament traits. Unlike the other types of children who gain emotional support through social interactions or reduce stress levels by participating in physical activities, the cautious children would not have proper channels to process their negative affectations and emotional distress because they are unwilling to join social interactions or activities requiring physical energy. Hence, the cautious children may overly depend on smartphones, which does not require direct social interaction and physical movement.

Further, problematic smartphone use of the cautious type of children may indicate the existence of other mental health issues or social dysfunctions related to low sociability and activity (Katz et al., 2011; Tang et al., 2020). Low sociability plays a significant role in the onset of depression (Elovainio et al., 2015). Anxiety and depression have strong links with problematic smartphone use (Matar Boumosleh and Jaalouk, 2017). The cautious type has the two core features of Type D personality: social inhibition and negative affectivity (Denollet, 2000, 2005). People with a Type D personality are susceptible to experiencing negative emotions across time and situations and are reluctant to share these emotions with others because of fear of rejection or disapproval, which encounter them being vulnerable to various physical and mental issues (Denollet, 2000; Nie et al., 2019). The cautious type found in the present study may be the prototype of the Type D personality. The cautious children maybe not only vulnerable to smartphone overdependence but to other internalizing mental health issues as well. Further studies should be conducted to identify the trajectory of the personality development and psychological adjustment of the cautious type of child.

In sum, this study identified three types of early temperament among Korean children using a population-based sample: reactive, social, and cautious. The link between early temperament types and smartphone overdependence was meaningful. The cautious children were regarded as the group most vulnerable to smartphone overdependence. Low sociability and activity traits observed in early years may be a precursor to later problematic smartphone use. Problematic smartphone use may be a maladaptive coping strategy of the cautious children (Squires et al., 2020). The cautious children need restorative activities to substitute for smartphone use (Nie et al., 2019), in which they can channel their emotional distress and reinforce social and physical skills without frustration or fear of rejection. Parents and early intervention practitioners need to help the cautious children build appropriate coping strategies. Developing healthy coping mechanisms will also minimize the risk of internalizing problems possibly associated with their socially and behaviorally inhibited temperamental nature. The current findings also highlight the unique value of the person-centered approach in temperament research (Gartstein et al., 2017; Dalimonte-Merckling and Brophy-Herb, 2019). When a variable-centered analysis was conducted using the same data set as the present study, among the three temperament traits at age 3, emotionality (β= 0.089, *p* = 0.003) was the only meaningful predictor of smartphone use at age 10. By adopting the person-centered approach, the researcher could produce more in-depth conclusions and enriching implications.

This preliminary study illustrates that predisposed temperamental types might exert longitudinal influences on smartphone use in childhood, which has rarely been introduced in the international academic discourse. The intersection of temperament types, a traditional topic in psychology research, and problematic smartphone use, an upcoming psychopathology issue, produced many interesting implications. The findings highlight that coordination of temperament traits may be an important factor to consider when examining problematic smartphone use of individuals. The high emotionality and high activity coordination, possessing the two most frequently discussed predictors of psychopathology, did not matter in problematic smartphone use. Rather children with socially and behaviorally inhibitive characteristics, known to be vulnerable to internalizing affective issues, had a greater risk of problematic smartphone use. These findings produce meaningful echoes regarding the nature of problematic smartphone use as psychopathology and how to treat and intervene. Future works should endeavor to clarify the position of problematic smartphone use in terms of the domain and hierarchy of psychopathology (Kotov et al., 2017). Also, inherited individual vulnerabilities associated with problematic smartphone use should be further explored and incorporated into prevention and intervention.

The findings from this study should be interpreted in light of the following limitations. First, in this study, conceptions of children’s temperament were constrained by the EAS of Buss and Plomin (1984). Also, mothers were the sole source of the information. Employing additional measures reflecting contemporary temperament research and multiple informers need to be incorporated in future research to extend our understanding of the link between temperament and smartphone usage. Second, the data collection of children’s smartphone usage solely depended on maternal ratings on a single measurement. Employing third-party observers who can rate children’s smartphone usage or incorporating children’s smartphone usage data from wireless telecommunication services will enhance the external validity of the current findings. Third, diverse circumstantial factors were not counted as contributors to children’s smartphone usage in this study. The roles of school settings, peer relationships, accessibility of free wireless internet, and parenteral behaviors need to be respected in further studies. Lastly, like many other longitudinal studies, the attrition of data was unavoidable. Among the 1,689 children whose temperament types were identified at age 3, only 1,296 data on children’s smartphone usage at age 10 were available. This data attrition may affect the results of this study.

## Data Availability Statement

Publicly available datasets were analyzed in this study. This data can be found here: Panel Study on Korean Children (https://panel.kicce.re.kr/pskc/index.do).

## Author Contributions

YK solely contributed to the whole process of manuscript development and submission.

## Conflict of Interest

The author declares that the research was conducted in the absence of any commercial or financial relationships that could be construed as a potential conflict of interest.

## Publisher’s Note

All claims expressed in this article are solely those of the authors and do not necessarily represent those of their affiliated organizations, or those of the publisher, the editors and the reviewers. Any product that may be evaluated in this article, or claim that may be made by its manufacturer, is not guaranteed or endorsed by the publisher.
